# Supplementary material for: COZOID: contact zone identifier for visual analysis of protein-protein interactions
Source: BMC Bioinformatics. 2018 Apr 6;19:125. doi: 10.1186/s12859-018-2113-6 (PMC5889581; doi:10.1186/s12859-018-2113-6)
Supplement: Supplementary file 2 — Software build. Executable binary file of the software tool. (ZIP 118,784 kb) [file 12859_2018_2113_MOESM2_ESM.zip › cozoid/java/docs/beansbinding-1.2.1-doc.zip!/org/jdesktop/beansbinding/AutoBinding.UpdateStrategy.html]

AutoBinding.UpdateStrategy (Beans Binding)


---


|  |  |  |
| --- | --- | --- |
| **Overview** \| **Package** \| **Class** \| **Use** \| **Tree** \| **Deprecated** \| **Index** \| **Help** | |  |
| **PREV CLASS**   **NEXT CLASS** | **FRAMES**    **NO FRAMES**     **All Classes** |  |
| SUMMARY: NESTED \| ENUM CONSTANTS \| FIELD \| METHOD | DETAIL: ENUM CONSTANTS \| FIELD \| METHOD |  |


---


## org.jdesktop.beansbinding Enum AutoBinding.UpdateStrategy

```
java.lang.Object
  java.lang.Enum<AutoBinding.UpdateStrategy>
      org.jdesktop.beansbinding.AutoBinding.UpdateStrategy
```

**All Implemented Interfaces:**: java.io.Serializable, java.lang.Comparable<AutoBinding.UpdateStrategy>

**Enclosing class:**: AutoBinding<SS,SV,TS,TV>

---

``` public static enum AutoBinding.UpdateStrategy extends java.lang.Enum<AutoBinding.UpdateStrategy> ```

An enumeration representing the possible update strategies of an
`AutoBinding`. See `AutoBinding's` class level
documentation for complete
details on the sync behavior for each possible update strategy.

---

**Enum Constant Summary**

`READ`
  
          An update strategy where the `AutoBinding` tries to keep the target
in sync with the source.

`READ_ONCE`
  
          An update strategy where the `AutoBinding` tries to sync the
target from the source only once, at bind time.

`READ_WRITE`
  
          An update strategy where the `AutoBinding` tries to keep both the
source and target in sync with each other.


| **Method Summary** | |
| --- | --- |
| `static AutoBinding.UpdateStrategy` | `valueOf(java.lang.String name)`             Returns the enum constant of this type with the specified name. |
| `static AutoBinding.UpdateStrategy[]` | `values()`             Returns an array containing the constants of this enum type, in the order they are declared. |

**Methods inherited from class java.lang.Enum**

`clone, compareTo, equals, finalize, getDeclaringClass, hashCode, name, ordinal, toString, valueOf`

**Methods inherited from class java.lang.Object**

`getClass, notify, notifyAll, wait, wait, wait`

**Enum Constant Detail**

### READ\_ONCE

```
public static final AutoBinding.UpdateStrategy READ_ONCE
```

:   An update strategy where the `AutoBinding` tries to sync the
    target from the source only once, at bind time.

---


### READ

```
public static final AutoBinding.UpdateStrategy READ
```

:   An update strategy where the `AutoBinding` tries to keep the target
    in sync with the source.

---


### READ\_WRITE

```
public static final AutoBinding.UpdateStrategy READ_WRITE
```

:   An update strategy where the `AutoBinding` tries to keep both the
    source and target in sync with each other.


**Method Detail**

### values

```
public static AutoBinding.UpdateStrategy[] values()
```

:   Returns an array containing the constants of this enum type, in
    the order they are declared. This method may be used to iterate
    over the constants as follows:

    ```
    for (AutoBinding.UpdateStrategy c : AutoBinding.UpdateStrategy.values())
        System.out.println(c);
    ```

    :   **Returns:**: an array containing the constants of this enum type, in the order they are declared

---


### valueOf

```
public static AutoBinding.UpdateStrategy valueOf(java.lang.String name)
```

:   Returns the enum constant of this type with the specified name.
    The string must match *exactly* an identifier used to declare an
    enum constant in this type. (Extraneous whitespace characters are
    not permitted.)

    :   **Parameters:**: `name` - the name of the enum constant to be returned. **Returns:**: the enum constant with the specified name **Throws:**: `java.lang.IllegalArgumentException` - if this enum type has no constant with the specified name: `java.lang.NullPointerException` - if the argument is null


---


|  |  |  |
| --- | --- | --- |
| **Overview** \| **Package** \| **Class** \| **Use** \| **Tree** \| **Deprecated** \| **Index** \| **Help** | |  |
| **PREV CLASS**   **NEXT CLASS** | **FRAMES**    **NO FRAMES**     **All Classes** |  |
| SUMMARY: NESTED \| ENUM CONSTANTS \| FIELD \| METHOD | DETAIL: ENUM CONSTANTS \| FIELD \| METHOD |  |


---
